# Quantification of T Cell Binding Polyclonal Rabbit Anti-thymocyte Globulin in Human Plasma with Liquid Chromatography Tandem-Mass Spectrometry

**DOI:** 10.1208/s12248-020-0419-6

**Published:** 2020-02-06

**Authors:** Mohsin El Amrani, Rick Admiraal, Lobke Willaert, Lysette J. C. Ebskamp-van Raaij, Amelia M. Lacna, C. Erik Hack, Alwin D. R. Huitema, Stefan Nierkens, Erik M. van Maarseveen

**Affiliations:** 1 Department of Clinical Pharmacy, Division of Laboratory Medicine and Pharmacy, University Medical Center Utrecht, Utrecht University, Heidelberglaan 100, Room no. D.00.318A, Internal post no. D.00.204, P.O. Box 85500, 3508 GA Utrecht, The Netherlands; 2 Center for Translational Immunology, University Medical Center Utrecht, Utrecht University, Utrecht, The Netherlands; 3 Princess Máxima Center for Pediatric Oncology, Utrecht, The Netherlands; 4 Department of Pharmacy & Pharmacology, Netherlands Cancer Institute, Amsterdam, The Netherlands

**Keywords:** Anti-thymocyte globulin, Immunoaffinity interaction, Jurkat T cell line, Liquid chromatography tandem-mass spectrometry, Polyclonal antibody, Quantification

## Abstract

The addition of rabbit anti-human thymocyte globulin (ATG) to the conditioning regimen prior to allogeneic hematopoietic cell transplantation has significantly reduced the risk of graft-*versus*-host disease (GvHD) and graft failure. However, ATG has a small therapeutic window. Overexposure of ATG post-HCT hampers T cell immune reconstitution and has been associated with increased relapse rates and viral reactivations, whereas underexposure has been associated with an increased incidence of GvHD, both of which lead to increased mortality. Therapeutic drug monitoring of T cell binding ATG plasma levels provides a means to optimize dosing for patients at high risk for graft failure to ensure timely T cell immune reconstitution and subsequently increase survival chances. This manuscript describes the first liquid chromatography tandem-mass spectrometry (LC-MS/MS) method to quantify the pharmacologically active fraction of polyclonal ATG in plasma. This was achieved through immunoaffinity purification of active ATG from plasma with Jurkat T cells. After the binding and washing, samples were eluted, denatured, and trypsin-digested. Signature peptides originating from the IgG constant chain were measured with LC-MS/MS. Critical method parameters were optimized, and the method was successfully validated following European Medicines Agency (EMA) guidelines. The method covered the therapeutic range of ATG and was validated at a lower limit of quantification (LLOQ) of 1 AU/mL with an overall CV and bias of 11.8% and − 2.5%, respectively. In conclusion, we developed a LC-MS/MS-based method to quantify active polyclonal rabbit ATG in human plasma. We suggest that this novel assay can be used to monitor and optimize dosing of ATG in clinical practice.

**Electronic supplementary material:**

The online version of this article (10.1208/s12248-020-0419-6) contains supplementary material, which is available to authorized users.

## INTRODUCTION

Allogeneic hematopoietic cell transplantation (HCT) is a potentially curative treatment strategy for both malignant and non-malignant life-threatening diseases such as leukemia, lymphoma, aplastic anemia, primary immune deficiencies, and inherited metabolic disorders (1). However, graft-*versus*-host disease (GvHD), with mortalities as high as 50%, poses a serious side effect with incidences ranging between 20 and 70%, depending on histocompatibility mismatches, the age of recipient, and the intensity of preparative regimes (2,3). GvHD is likely caused by the transplanted donor T cells recognizing major and minor histocompatibility complex proteins on the recipient antigen-presenting cells (3). Prophylaxis with polyclonal anti-thymocyte globulin (ATG) which targets different antigens expressed on, *e.g.*, T cells, B cells, natural killer cells, and dendritic cells lead to depletion of these cells from the host blood and peripheral lymphoid tissues (4). Though ATG is used as a rescue therapy for acute rejection in solid organ transplantation, its main application is in hematology to treat and prevent acute and chronic GvHD following HCT in patients with hematologic cancers (4,5). However, the overall survival remains similar between ATG-treated and ATG-untreated patients due to increased risk of relapse and infections in ATG-treated patients (6–9). This outcome is defined by the delicate balance in timing of ATG exposures, where a high ATG exposure pre-HCT is associated with reduced GvHD and graft failures, but high exposures post-HCT are associated with increased relapse rates and reduced survival chances in patients with viral reactivations and GvHD. The presence of lytic levels of ATG post-HCT leads to poor reconstitution of donor T cells which in turn would limit graft *versus* tumor effect leading to increased risk of a relapse and a reduced control of viruses and regulation of GvH-activity. European Society of Bone Marrow transplantation (EBMT) has historically recommended dosing ATG at a rate of 7.5 mg/kg over 3 days starting from day − 3 pre-HCT in adults. Our group has shown that this dosing leads to overexposure in a majority of patients since dosing is close to graft infusion, and absolute lymphocyte counts (ALS), the most important determinant of ATG clearance at patient’s body weight exceeding 40 kg, is not taken into account (6,10). Furthermore, to allow for improved ATG exposure, an alternative dosing regimen starting at day − 9 and dosing for 4 days based on ALC values and weight has been proposed. However, optimum ATG dosing of high-risk patients such as those with chronic granulomatous disease (CGD) or hemophagocytic lymphohistiocytosis (HLH) remains difficult possibly because of large T cell pools in tissues. In these patients, therapeutic drug monitoring (TDM) of free T cell binding (active) ATG may improve CD4^+^ immune reconstitution and prevention of graft failure (11).

Currently, fluorescent-activated cell sorting (FACS) is used for the quantification of active ATG in plasma (12). Liquid chromatography tandem-mass spectrometry (LC-MS/MS) is increasingly used in quantitative proteomics, because of its superior selectivity and linear dynamic range, as compared with ligand binding assays (13). The quantification of therapeutic monoclonal antibodies (mAbs) in preclinical (14–17) and in clinical samples (18–22) using LC-MS/MS has been reported. MAbs are generally quantified by their unique signature peptide originating from either the variable region for clinical samples or sometimes from the constant region for preclinical samples (23). However, to the best of our knowledge, the quantification of polyclonal animal-based therapeutic antibodies in human plasma has not previously been reported. Here, we describe the application of LC-MS/MS to quantify therapeutic polyclonal rabbit antibodies, ATG, in human plasma samples based on constant region signature peptides.

## MATERIALS AND METHODS

### Chemicals and Reagents

Rabbit ATG was obtained from Sanofi Genzyme (Cambridge, MA, USA) as a 25-mg lyophilized powder which was dissolved in ultra-pure LC-MS-grade water to a final concentration of 5 μg/μL. Aliquots were stored at − 80°C until further use. Jurkat T cells were grown as previously described (24). Internal standard (IS) stable isotopically labeled (SIL) peptide “LSVPTSEWQ(R 13C_6_,15N_4_)” was obtained from Pepscan Presto BV (Lelystad, The Netherlands). Bovine serum with reference number 26010-074 was obtained from Life Technologies™ (Carlsbad, CA, USA). Human K2 EDTA plasma and serum were obtained from volunteers at the UMCU (Utrecht, The Netherlands). TPCK-Trypsin was supplied by Thermo Scientific (Waltham, MA, USA) as a lyophilized powder and was dissolved in acetic acid (50 mM) to a concentration of 10 μg/μL, aliquoted in Eppendorf LoBind Microcentrifuge tubes and stored at − 80°C. All other chemicals, reagents, and LC-MS-grade mobile phase solvents were obtained from Sigma-Aldrich (Saint Louis, MO, USA).

### Preparation of Standards, Internal Standard, and Quality Control Samples

Since only a fraction of ATG in the stock standard is capable of specifically binding to T cells (25), a conversion to arbitrary units (AU) was necessary. Arbitrary units are used when the real concentration is unknown. In this instance, the stock solution contains a total IgG fraction of which an unknown percentage specifically binds to T cells. Here, the ATG stock standard of 5 mg IgG per mL was set as 5000 AU active ATG per mL as was previously described (6).

ATG working solution of 512 AU/mL was prepared by pipetting 64 μL of ATG stock together with 561 μL pooled plasma from five healthy donors in a LoBind Eppendorf tube. The highest standard solution of 32 AU/mL was prepared by combining 20 μL working solution with 300 μL pooled EDTA plasma.

Remaining standards 1, 2, 4, 8, and 16 AU/mL were freshly prepared from the highest standard 32 AU/mL through serial dilution in pooled plasma. The internal standard SIL peptide solution “LSVPTSEWQ(R 13C_6_,15N_4_)” at a concentration of 50 ng/mL was prepared in 0.05% Zwittergent™ 3-16 and 1% formic acid in water. Quality control samples (QCs) at lower limit of quantification (LLOQ) (1 AU/mL), QC low (3 AU/mL), QC med (6 AU/mL), and QC high (14 AU/mL) were prepared in pooled plasma from a separate batch of healthy donors to allow for matrix variations between the standards and the controls. Aliquots were stored at − 80°C.

### Instrumentation and Chromatographic Conditions

A HulaMixer™ from Thermo Fisher (Waltham, MA, USA) was used during immunoaffinity interaction. ThermoMixer C from Eppendorf™ (Hamburg, Germany) was used for denaturation and digestion. Centrifuge used was the Rotina 380R with a 96-well plate rotor from Hettich (Kirchlengern, Germany). All measurements were performed on a Vanquish LC coupled to a TSQ Altis mass spectrometer, Thermo Fisher (Waltham, MA, USA). The analytical column was Acquity UPLC™, BEH, C18, 2.1 × 150 mm, 1.7-μm particle size, Waters Corporation (Milford, MA, USA); the guard column was the SecurityGuard column ULTRA C18, 2.1 mm, Phenomenex (Torrance, CA, USA). Both were maintained at 50°C. The mobile phases were (a) 0.1% formic acid in water and (b) 0.1% formic acid in acetonitrile. The LC gradients in minutes per percentage of mobile phase B were 0.0 (min)/15 (% B), 4.5/30, 4.6/80, 5.5/80, 5.6/15, and 7/15. The flow rate was 0.45 mL/min, injection volume was 20 μL, and the run time was 7 min. The mass spectrometer was operated in positive mode with spray voltage of 2 kV, ion transfer tube temperature 400°C, vaporizer temperature 350°C, aux gas pressure 20 arb, sheath gas pressure 50 arb, and CID Gas 2.5 mTorr. The precursor ion and product ion settings are listed in Table I for ATG and for the SIL internal standard.

**Table I:** TSQ Altis Mass Spectrometry Conditions for SRM Transitions for the Signature Peptide Liberated from ATG After Digestion with Trypsin and the Internal Standard Stable Isotopic-Labeled Peptide

Peptide sequence	Used as	RT^a^ (min)	Precursor (m/z)	Product (m/z)	Production type	CE^b^ (V)	RF^c^ (V)	Dwell time (ms)
LSVPTSEWQR	Quantifier	2.9	601.81	903.43	Y7	20	70	250
LSVPTSEWQR[13C_6_,15N_4_]	IS^d^	2.9	606.81	913.43	Y7	20	70	50
VVSTLPIAHQDWLR	Qualifier	4.1	817.95	1135.60	Y9	30	100	300

^*a*^*RT retention time*

^*b*^*CE collision energy*

^*c*^*RF radio frequency lens*

^*d*^*IS internal standard*

### LC-MS/MS Sample Preparation

A selective sample purification method, on the basis of immunoaffinity interaction, was used to capture the polyclonal active ATG fraction only. In brief, 250 μL Jurkat T cell suspension (4 × 10^6^ cells/mL) in phosphate-buffered saline (PBS buffer) and 1% human serum albumin (HSA) were pipetted in a LoBind 500-μL 96-deep-well plate. Subsequently, 10 μL standard, QC, or plasma sample was added and incubated for 2 h at room temperature. Cells were centrifuged (500*g* for 5 min) and washed twice with 250 μL PBS buffer. Each time, the pellet was resuspended and washed through vortex mixing at 1200 RPM for 1 min and centrifugation at 500*g* for 5 min. The pellet was finally resuspended in 135 μL water with a vortex at 1200 RPM for 1 min. Hereafter, 15 μL IS solution containing SIL peptide (50 ng/mL in 1% formic acid, 0.05% Zwittergent™ in water) was added and mixed for a further 5 min, eluting bound ATG from the Jurkat T cells. The mixture was centrifuged for 5 min at 500*g*, and 100 μL supernatant was pipetted into a 500-μL LoBind 96-well plate. Here ATG was heat-denatured at 80°C for 30 min. Then, the solution was cooled to room temperature, and 10 μL 1 M TRIS (unbuffered) was added to neutralize the solution. Thereafter, 10 μL trypsin (1 μg/μL) was added and the mixture was digested at 37°C for 1 h. Finally, 20 μL acetonitrile with 10% formic acid was added and mixed to stop the trypsin activity and dissolve the peptides. Of this solution, 20 μL was injected and analyzed on the LC-MS/MS.

### Signature Peptide Selection

ATG consists of polyclonal rabbit gamma immune globulins. Therefore, for the selection of the signature peptides used for quantification and qualification, we focused on peptides originating from the constant chains of rabbit IgG and that are not endogenous to humans. Furthermore, the selected peptides should contain stable amino acids and be between 6 and 20 amino acids long. Using the amino acid sequence of rabbit IgG constant region with locus 2VUO_B (FASTA file obtained from The National Center for Biotechnology Information, https://www.ncbi.nlm.nih.gov/), a selection of *in silico* tryptic peptides was made based on predicted stability and length. Identified peptides were matched against the human genome proteins (https://blast.ncbi.nlm.nih.gov/Blast.cgi), resulting in a list of potential tryptic peptide candidates. Then, 30 μL ATG stock solution (5 μg/μL) in 100 μL Tris buffer (100 mM, pH 8.5) was denatured, digested, and measured. A shallow LC-MS/MS gradient 0–40% organic in 10 min was used with MS operating in product ion scan mode using doubly and triply charged precursors in two consecutive runs. Two peptides with the highest signals and lowest noise were chosen for further optimization.

### Effect of Incubation Time on Signal Intensity

ATG and Jurkat T cell incubation time was optimized using 10 μL ATG standard at 32 AU/mL and 250 μL Jurkat T cell suspension at 2 × 10^6^ cells/mL. The “LC-MS/MS Sample Preparation” section described above was followed with the following modifications, LoBind Eppendorf tubes were used and an incubation time of 1, 2, 3, and 4 h was maintained in triplicate. Upon completion of each time point, samples were centrifuged, unbound fraction was decanted, and the pellet was washed. All samples were digested and measured simultaneously.

### Effect of Jurkat T Cell Number on Signal Intensity

The optimum number of Jurkat T cells used in relation to the highest standard 10 μL of 32 AU/mL was empirically determined. Different volumes 12.5–25–50–100–200–250 μL of Jurkat T cells suspension 10 × 10^6^ cells/mL were pipetted in triplicates in LoBind 96-deep-well plate, and final volume was adjusted to 250 μL with PBS. Then, 10 μL of the highest standard 32 AU/mL was added to the cells and allowed to incubate for 2 h. The samples were subsequently treated as described, and signal intensity of the signature peptides and the IS was recorded.

### Optimization of Digestion Time

In this experiment, the time required for optimum digestion of a denatured ATG standard solution (32 AU/mL) was investigated. In 18 LoBind Eppendorf tubes, 10 μL ATG standard solution 32 AU/mL was mixed with 80 μL water and 10 μL SIL IS solution (50 ng/mL, 1% formic acid, 0.05% Zwittergent™ 3-16 in water). The tubes were placed on the thermomixer C at 80°C for 1 h. After the samples were cooled to room temperature, 10 μL 1 M Tris buffer was added to raise the pH to 8.5. Finally, 10 μL trypsin solution (1 μg/μL) was added and samples were digested in triplicates during 1, 2, 3, 4, and 5 h and overnight after which trypsin activity was stopped though the addition of 20 μL 10% formic acid in acetonitrile.

### Matrix Effect

Matrix effect on MS-ionization and matrix-analyte interactions was investigated in this experiment. QC low (3 AU/mL) and QC high (14 AU/mL) were prepared in PBS, human serum (HS), human plasma (HP), and bovine serum (BS). Samples were purified with Jurkat T cells and analyzed in fivefolds following the “LC-MS/MS Sample Preparation” section described above.

### Validation

The method was validated following European Medicines Agency (EMA) guidelines for bioanalytical method validation (26).

### Jurkat T Cell Robustness Test

Thirty EDTA plasma samples from patients treated at the UMCU with ATG have been measured with the LC-MS/MS method on two separate days. The samples were drawn in 4 mL K2 EDTA tubes, centrifuged at 4000 RPM and stored in − 80°C prior to analysis. Samples were selected based on the concentration range of active ATG. Three ranges were defined, low (0–2 AU/mL), med (2–7 AU/mL), and high (7–30 AU/mL), and 10 samples from each range were selected. The first run was made with a fresh batch of Jurkat T cells. The second was from the same batch as the first but harvested a week later.

The use of anonymized remnant material drawn as part of the treatment protocol and with patient’s informed consent was according to the University Medical Center Utrecht policy and ethical standards.

## RESULTS

### Signature Peptide Selection

*In silico* digestion of rabbit IgG constant chain locus 2VUO_B produced 12 tryptic peptides with chain length between 6 and 20 amino acids long (Supplement materials, Table S1). Four peptides contained the unstable amino acids asparagine, methionine, and cysteine and were thus omitted. The remaining peptides were compared with the human Swiss-Prot database using blast. This search showed that three peptides were also endogenous to humans by having 100% match for both the query cover and identification and were thus dismissed. The remaining peptides were screened for signal intensity and chromatographic interference. Two peptides LSVPTSEWQR and VVSTLPIAHQDWLR were found to have the highest signal intensity and the lowest chromatographic interference and were thus chosen as signature peptides.

### Method Development

ATG is a mixture of rabbit non-specific, random IgG, and T cell binding IgG. Plasma levels of the latter fraction correlate with therapeutic response (11,27). Therefore, a selective sample purification method was set up to specifically quantify the free T cell binding (active) ATG faction. Jurkat T cells, an immortalized human T lymphocyte line, were used to purify active ATG from plasma. After sample purification, active ATG was eluted with 0.1% formic acid containing SIL internal standard and Zwittergent™ 3-16. As an internal standard, a stable isotopically labeled peptide with the sequence LSVPTSEWQR[13C6,15N4] was used to correct for volume differences and other LC-MS/MS-related variations such as injection, ionization suppression, fragmentation, and signal drift. Zwittergent™ 3-16, an MS compatible synthetic zwitterionic detergent, was used to reduce van der Waals interaction within and between proteins, thus promoting solubility. After elution, active ATG was denatured at 80°C and trypsin-digested according to our previous work (18). The internal standard SIL peptide was measured together with two ATG tryptic peptides originating from the constant chain (Table I).

### Effect of Incubation Time on Signal Intensity

To assess the incubation time required for optimum immunoaffinity, 10 μL of the highest ATG standard of 32 AU/mL was incubated with 250 μL Jurkat T cell suspension 2 × 10^6^ cells/mL for up to 4 h.

**Fig. 1:**
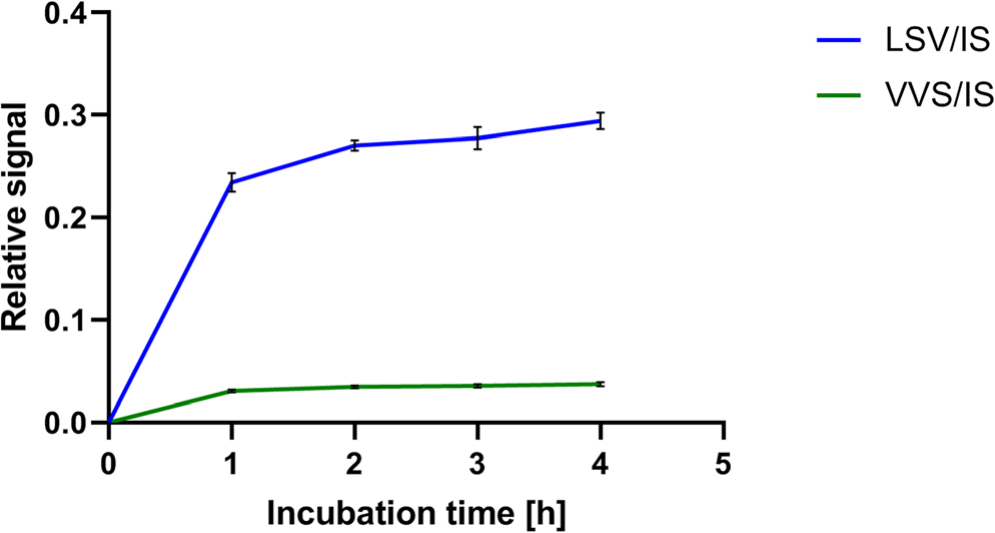
Effect of incubation time (*x*-axes in hours) on relative signal intensity (*y*-axes) ratio signature peptide LSV and VVS divided by internal standard (IS). Results represent mean and SD of *n* = 3

Figure 1 shows that at 2-h incubation, a stable plateau was reached. Only a slight signal increase < 10% was observed between the 2- and 4-h time points. Therefore, a 2-h incubation period was used in further experiments. As the signal intensity of LSV was approximately six times higher compared with that of VVS signature peptide, LSV was chosen as the quantifier and VVS as qualifier peptide.

### Effect of Jurkat T Cell Number on Signal Intensity

**Fig. 2:**
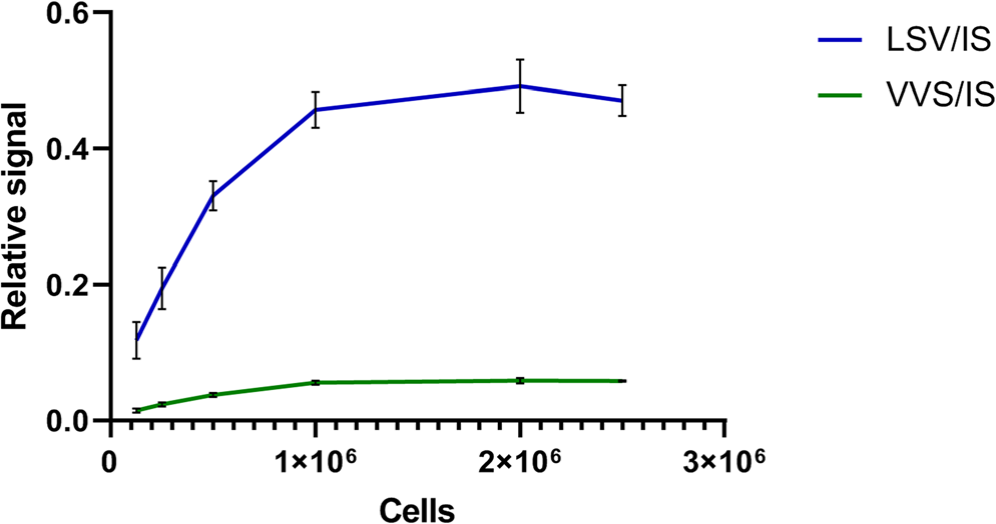
Effect of the number of cells in 96-well plate (*x*-axes) on the relative signal intensity of the highest ATG standard 32 AU/mL (*y*-axes). Incubation time with Jurkat T cells was 2 h. Results represent mean and SD of *n* = 3

The optimal number of Jurkat T cells needed to capture active ATG from 10 μL of the highest standard 32 AU/mL was established. As can be seen from Fig. 2, both signature peptides (LSV and VVS) reached a plateau when 1 million cells were used. Furthermore, the coefficient of variation was smaller when more cells were used. After centrifugation, the large compact pellets were more firmly attached to the 96-well plate surface; thus, minimal sample loss incurred during the washing steps.

### Optimization of Digestion Time

Optimum digestion time was investigated with ATG spiked in the same TRIS buffer solution as described in “LC-MS/MS Sample Preparation.” Triplicates were digested during 1, 2, 3, 4, and 5 h and overnight.

**Fig. 3:**
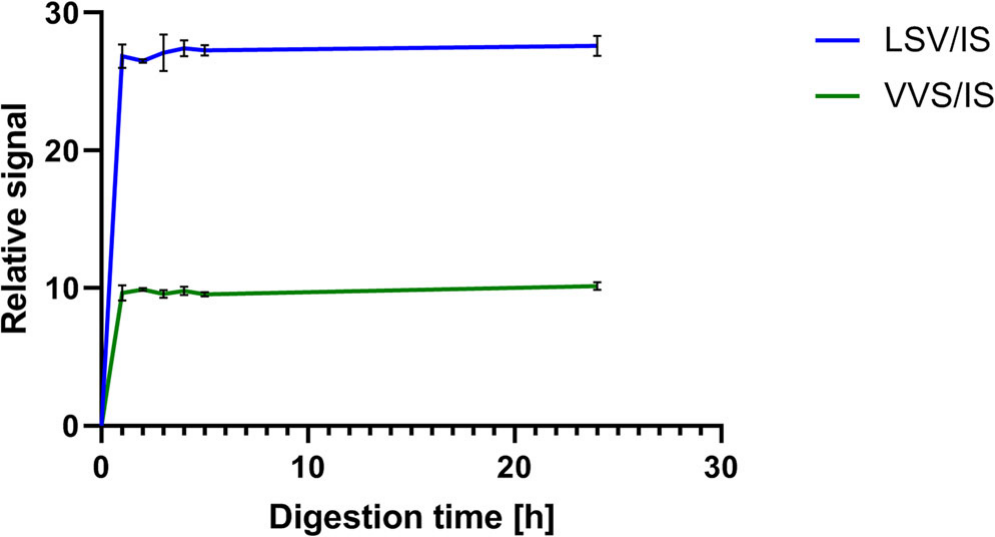
Effect of digestion time (*x*-axes in hour) on the relative signal intensity of the highest ATG standard 32 AU/mL. Results represent mean and SD of *n* = 3

Data obtained shows that 1-h digestion provides similar signal intensity as overnight digestion (Fig. 3).

### Matrix Effect

Cross-reactivity of ATG with matrix components such as endogenous immunoglobulins, coagulation factors, or albumin could lead to low T cell binding ATG recoveries. Furthermore, matrix could also interfere with MS-ionization, leading to signal suppression and thus resulting in loss of sensitivity. To correct for this, an additional sample cleanup would be required to eliminate the interfering matrix component.

**Fig. 4:**
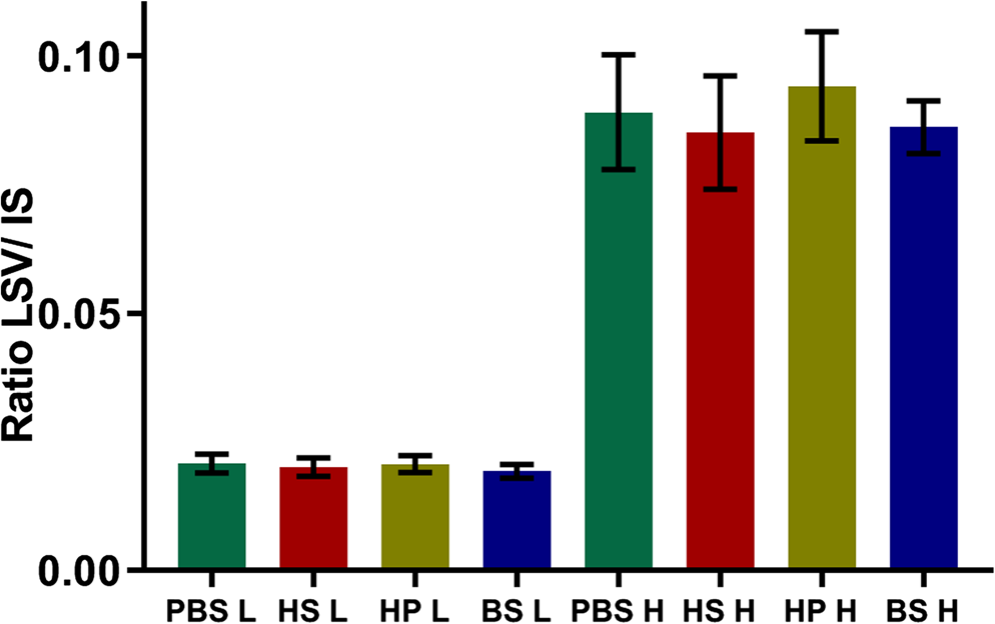
Comparison of relative signal intensity (*y*-axes) obtained from various matrixes (PBS = phosphate-buffered saline, HS = human serum, HP = human plasma, BS = bovine serum) ATG spiked at low (3 AU/mL) and high (14 AU/mL) concentrations (*x*-axes). Results represent mean and SD of *n* = 5

**Fig. 5:**
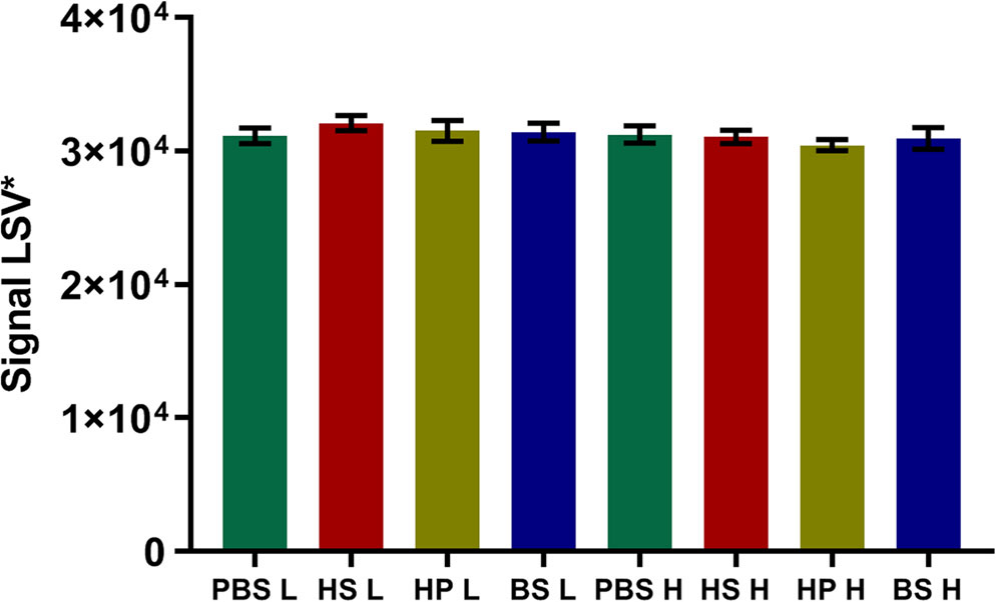
Comparison of internal standard signal intensity (*y*-axes) obtained from various matrixes. Results represent mean and SD of *n* = 5

As can be seen from spiking experiments shown in Fig. 4, there are no significant differences between the various matrices tested in both the low and high concentration range. Therefore, analysis can be performed in both serum or EDTA plasma. Furthermore, differences in ionization were tested by comparing the internal standard (IS) signal originating from ATG spiked in PBS with the IS signal obtained from the various sample matrices (Fig. 5). No ionization suppression or enhancement was observed between the various matrices tested and PBS, for both low and high ATG-spiked concentrations.

### Validation

**Fig. 6:**
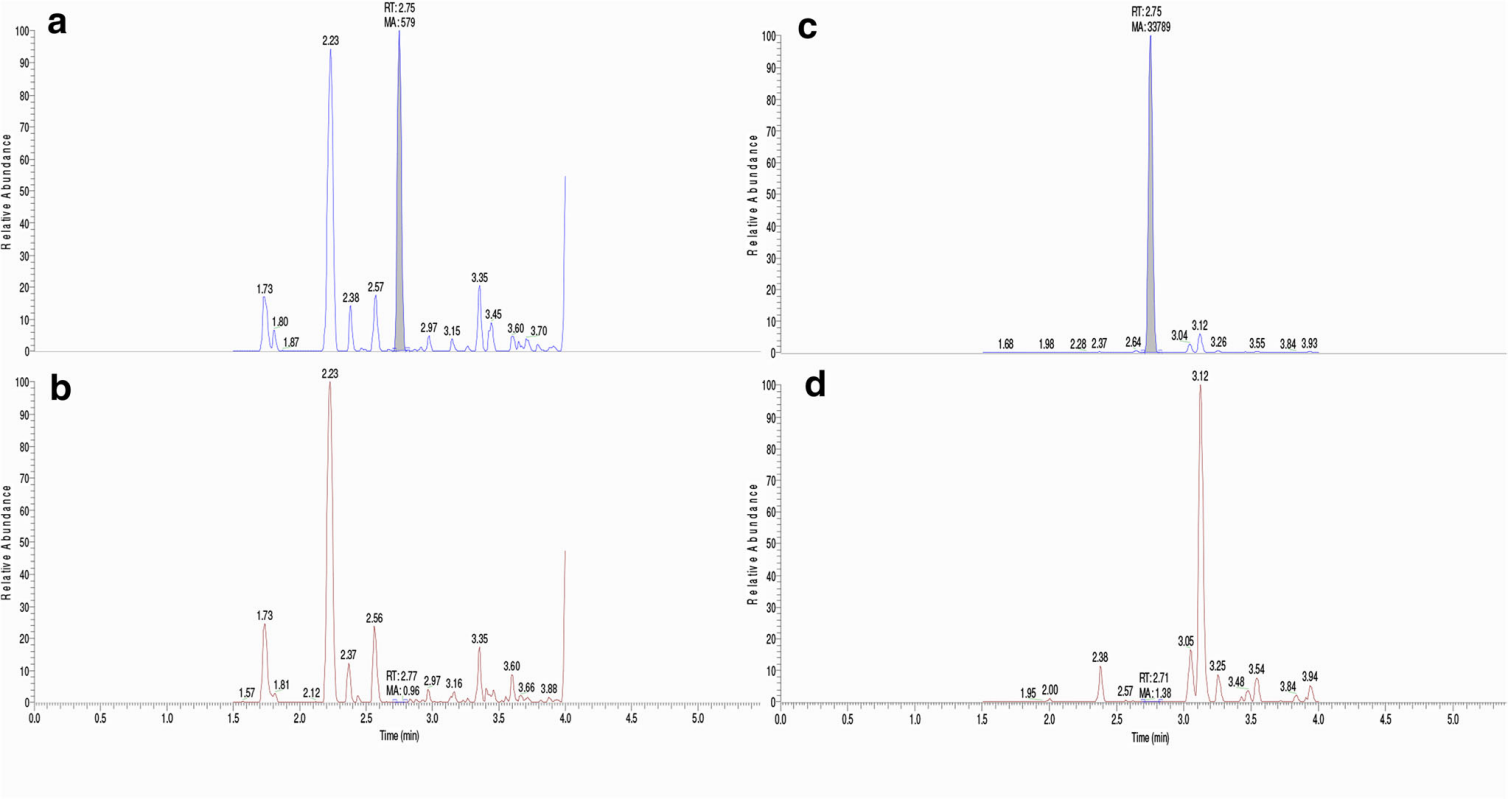
Left side, LC-MS/MS chromatogram of ATG standard 1 AU/mL (**a**) and chromatogram of negative human plasma containing IS (**b**) both measuring LSVPTSEWQR peptide with SRM transition 601.81 ➔ 903.43. Right side, chromatogram of internal standard (**c**) and chromatogram of blank (**d**) measuring the stable isotopically labeled peptide LSVPTSEWQR[13C_6_,15N_4_] with SRM transition 606.81 ➔ 913.43

The LLOQ for the quantifier peptide LSV was validated by comparing the signal at LLOQ level (1 AU/mL) to the noise obtained from a negative human plasma sample (Fig. 6a, b). Here, a signal-to-noise ratio of around 500 was obtained, which was significantly above the EMA guidelines of S/*N* > 5. This could potentially allow for an even lower detection limit if needed for future studies. Furthermore, blank human plasma did not contain any detectable peak at the retention time of the internal standard, thus ensuring a high selectivity (Fig. 6c, d). Calibration curve was linear between 1 and 32 AU/mL with *R*^2^ > 0.999. Carry over was 0.02% of LLOQ signal, and overnight auto sampler stability was evaluated by reinjecting LLOQ, QC low, and QC high after 5 days; the difference between results were < 10% for all values which is within guidelines of 15% (supplement materials, Table S2). Freeze/thaw stability of ATG was validated during three freeze/thaw cycles in fivefold using QC low and QC high. Both QC low and QC high were stable during the 3 cycles; bias and CV did not exceed the threshold of 15% (supplement materials, Table S3). Finally, accuracy and precision were validated during 3 days with LLOQ, QC low, QC medium, and QC high measured in fivefold (Table II, Table S4). Both parameters were in concordance with guidelines. An additional matrix effect experiment was carried out by spiking plasma from 6 volunteers at QC low and QC high concentration. Back-calculated concentrations were all within 15% bias of the true value (Table III).

**Table II:** Accuracy and Precision Validation Data for ATG QC’s at LLOQ, Low, Medium, and High Levels. Within-Run Data Were Based on 5 Replicates and Between-Run Data on 3 Different Days. Data Based on the Quantifier Peptide LSV Measuring 601.81 ➔ 903.43 Transition

QC	Precision (% CV)			Accuracy (% bias)
	Within-run	Between-run	Overall	Overall
LLOQ	10.7	5.0	11.8	− 2.5
Low	7.6	7.5	10.7	0.9
Med	7.7	2.5	8.1	− 3.1
High	5.2	9.0	10.4	− 2.9

*QC*, quality control; *LLOQ*, lower limit of quantification

**Table III:** Matrix Effect Test. Six Human Plasma Samples Spiked with ATG at QC Low (3 AU/mL) and QC High Levels (14 AU/mL)

Sample no.	Measured (AU/mL)	Bias	Measured (AU/mL)	Bias (%)
1	2.99	− 0.3	13.64	− 2.6
2	2.93	− 2.3	13.03	− 6.9
3	2.86	− 4.7	14.38	2.7
4	3.25	8.3	13.91	− 0.6
5	2.80	− 6.7	12.87	− 8.1
6	2.56	− 14.8	12.60	− 10.0

### Jurkat T Cell Robustness Test

**Fig. 7:**
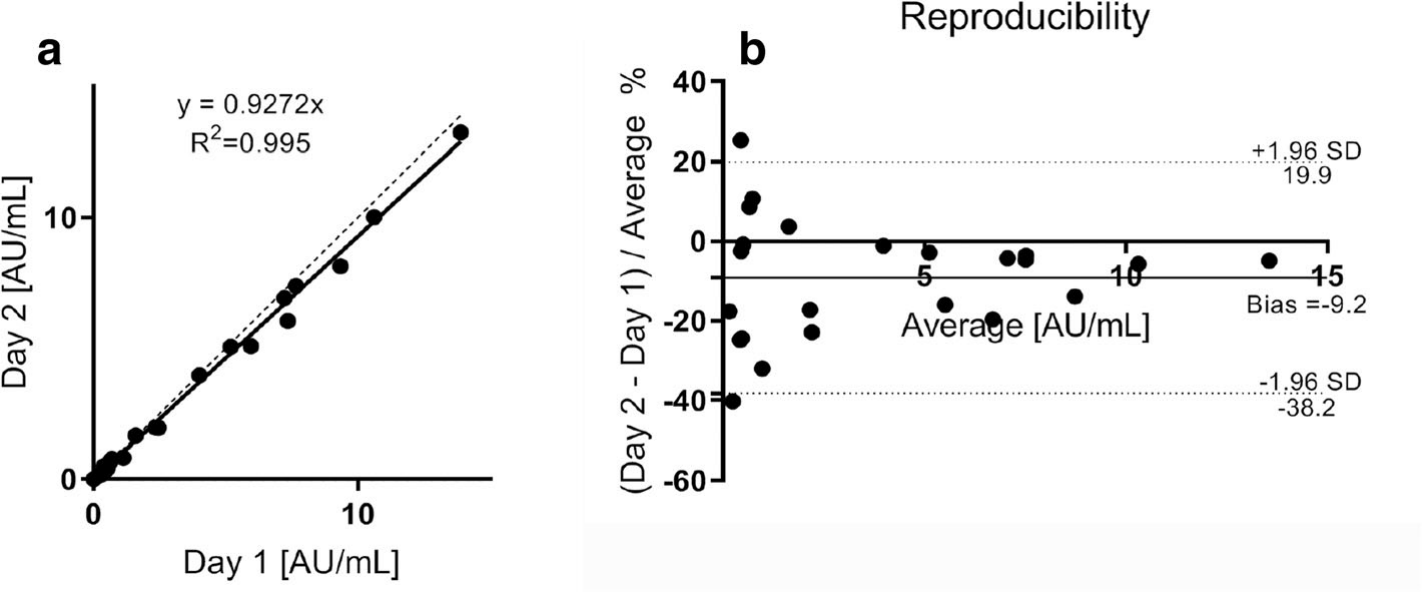
Pearson’s regression (**a**) and Bland-Altman plot (**b**) of reproducibility data obtained during the analysis of active ATG in patients EDTA plasma samples on two separate days *n* = 30

Plasma samples from 30 patients treated with ATG have been analyzed with a freshly made Jurkat T cell batch and the same batch harvested 1 week later for the second day run. Pearson’s regression (Fig. 7a) shows that reproducible results were obtained (*r*^2^ = 0.995). Bland-Altman plot (Fig. 7b) indicates an overall bias of − 9% which is within acceptance criteria of 15%. At the lower concentration scale, we see a larger variability but this is to be expected since values at the LLOQ range (1 AU/mL) are allowed a bias ≤ 20%. Results obtained from the quantifier ion measurement were compared with those from the qualifier ion, and a good correlation was found (supplement materials, Fig. S1). The results from the lower end 0–3 AU/mL were not as strong as the higher end due to the higher detection limit of the qualifier peptide. This data shows that patients treated with ATG can successfully be monitored in the relevant concentration range with LC-MS/MS.

## CONCLUSION/DISCUSSION

Here we describe the first LC-MS/MS method to quantify active ATG. LC-MS/MS allows for increased selectivity by directly measuring the signal intensity of LSVPTSEWQR and VVSTLPIAHQDWLR peptides, which originate from the constant chain and are exclusively present in rabbit IgG. The qualifier peptide VVS provided a means to check the results obtained from the quantifier peptide LSV as to ascertain that no isobaric interferences were present. Differences in results between VVS and LSV greater than > 15% are a sign of possible matrix interference and thus warrant further investigation. This method allows samples to be quantified for research purposes and routine therapeutic drug monitoring. Furthermore, due to its linear dynamic range 1–32 AU/mL, it allows quantification to be performed without the need of multiple sample dilutions as in the case with FACS methods. The assay does not depend on fluorescent tagged antibodies for detection and is easy to perform requiring only two washing steps after incubation. The use of 96-well plate format enables high-throughput analysis and the small sample volume required for analysis 10 μL is less invasive for the younger patients.

The limitation of the LC-MS/MS method is the introduction of the stable isotopically labeled internal standard in conjunction with the elution step following the immunoaffinity capture. This means that the internal standard cannot correct for loss during the binding and washing steps. However, since the pallet remains tightly bound to the plate after centrifugation, the within-run error was found to be < 15% CV. Furthermore, by performing duplicate analysis of the sample, it is possible to monitor the efficiency of this step.

The therapeutic window for ATG has been described in pediatric patients receiving bone marrow and cord blood after myeloablative conditioning and adult patients receiving peripheral blood stem cells after reduced intensity conditioning. Still, the therapeutic window remains to be determined in more settings, including T cell–depleted and haplo transplants.

Furthermore, the proposed population pharmacokinetics models for children and adults adequately predict concentration-time curves (6). There is however some unexplained variability in clearance, more profound in adults compared with children. This variability can be eliminated by performing TDM.

TDM can also be used in patients at high risk for graft failure and GvHD, where more extreme exposures to ATG may be needed (11). By monitoring and optimizing the concentration of active ATG, immune reconstitution of high-risk patients can be improved and graft failure can be prevented both of which have been associated with increased overall survival.

## Electronic supplementary material

ESM 1 (DOCX 79 kb)
