# Supplementary material for: Quantification of T Cell Binding Polyclonal Rabbit Anti-thymocyte Globulin in Human Plasma with Liquid Chromatography Tandem-Mass Spectrometry
Source: AAPS J. 2020 Feb 6;22(2):43. doi: 10.1208/s12248-020-0419-6 (PMC7005072; doi:10.1208/s12248-020-0419-6)
Supplement: Supplementary file 1 — (DOCX 79 kb) [file 12248_2020_419_MOESM1_ESM.docx]

**Supplementary Material associated with:**

**Quantification of T-cell binding polyclonal rabbit anti-thymocyte globulin in human plasma with liquid chromatography tandem mass-spectrometry**

Mohsin El Amrani^1^, Rick Admiraal^2,4^, Lobke Willaert^1^, Lysette J.C. Ebskamp-van Raaij^2^, Amelia M. Lacna^2^, C. Erik Hack^2^, Alwin D.R. Huitema^1,3^, Stefan Nierkens^2,4*^, Erik M. van Maarseveen^1*†^

^1^Department of Clinical Pharmacy, Division of Laboratory Medicine and Pharmacy, University Medical Center Utrecht, Utrecht University, Utrecht, The Netherlands.

^2^Center for Translational Immunology, University Medical Center Utrecht, Utrecht University, Utrecht, The Netherlands.

^3^Department of Pharmacy & Pharmacology, Netherlands Cancer Institute, Amsterdam, The Netherlands

^4^Princess Máxima Center for Pediatric Oncology, Utrecht, The Netherlands.

* Both authors contributed equally to this manuscript

Corresponding Author

† E-Mail: [E.M.vanMaarseveen@umcutrecht.nl](mailto:E.M.vanMaarseveen@umcutrecht.nl).

| Table S1. Peptides with amino acids (6<n<20) obtained after in-silico digestion of rabbit constant chain locus 2VUO_B. Results for query cover and identification percentages were obtained from pBlast using human library from the Swiss-Prot database | | | | |
| --- | --- | --- | --- | --- |
| Sequence | Mass | Stability | Query cover | Identification |
| EELSSR | 720.35 | Yes | 100% | 100% |
| AEDNYK | 739.33 | Yes | 83% | 100% |
| GQPLEPK | 768.43 | Yes | 85% | 100% |
| TARPPLR | 810.49 | Yes | 85% | 100% |
| DTLMISR | 835.43 | No | 100% | 100% |
| ALPAPIEK | 838.50 | Yes | 100% | 100% |
| VYTMGPPR | 920.47 | No | 75% | 83% |
| EQQFNSTIR | 1122.55 | No | 100% | 78% |
| LSVPTSEWQR | 1202.62 | Yes | 80% | 75% |
| VVSTLPIAHQDWLR | 1634.90 | Yes | 92% | 69% |
| TTPAVLDSDGSYFLYSK | 1863.90 | Yes | 100% | 88% |
| CPPPELLGGPSVFIFPPKPK | 2120.16 | No | 100% | 90% |

**Autosampler stability**

| Table S2. Reinjection of standards and QC samples 5 days after the first injection. Samples stored in a cooled, 10°C, autosampler. | | | |
| --- | --- | --- | --- |
| Sample | Measured  Day 1  [AU/mL] | Measured  Day 5  [AU/mL] | Bias  [%] |
| LLOQ 1 | 0.81 | 0.76 | -6.4 |
| LLOQ 2 | 0.8 | 0.87 | 8.4 |
| QC Low | 2.81 | 2.82 | 0.4 |
| QC Low | 2.9 | 2.78 | -4.2 |
| QC High | 12.59 | 12.37 | -1.8 |
| QC High | 12.77 | 12.47 | -2.4 |

**Freeze/thaw stability**

| Table S3. Freeze/ thaw stability tested with three freeze/thaw cycles and analyzed in five-fold. | | | | | | | | |
| --- | --- | --- | --- | --- | --- | --- | --- | --- |
| Nominal value  [AU/mL] | Day | #1 | #2 | #3 | #4 | #5 | CV  [%] | Bias  [%] |
| QC Low | 1 | 2.91 | 2.73 | 2.88 | 2.56 | 2.70 |  |  |
| 3 | 2 | 3.32 | 3.11 | 2.79 | 2.87 | 2.80 | 6.9 | 3.0 |
|  | 3 | 3.19 | 3.07 | 2.87 | 3.04 | 2.84 |  |  |
| QC High | 1 | 12.25 | 12.30 | 11.97 | 12.52 | 12.22 |  |  |
| 14 | 2 | 16.00 | 14.40 | 13.97 | 14.39 | 14.41 | 8.7 | 4.4 |
|  | 3 | 14.14 | 13.66 | 12.97 | 13.15 | 12.32 |  |  |

**Inter and intra-assay validation data**

| Table S4. Inter and intra-assay precision and accuracy tested during three days in five-fold | | | | | | | | |
| --- | --- | --- | --- | --- | --- | --- | --- | --- |
| Nominal value  [AU/mL] | Day | #1 | #2 | #3 | #4 | #5 | CV  [%] | Bias  [%] |
| LLOQ | 1 | 1.02 | 0.90 | 0.86 | 0.85 | 0.86 |  |  |
| 1 | 2 | 1.27 | 0.87 | 1.00 | 0.97 | 0.92 | 11.5 | 2.5 |
|  | 3 | 1.08 | 0.93 | 1.07 | 1.01 | 1.01 |  |  |
| QC Low | 1 | 2.91 | 2.73 | 2.88 | 2.56 | 2.70 |  |  |
| 3 | 2 | 3.37 | 3.16 | 3.26 | 3.58 | 2.87 | 9.9 | -0.9 |
|  | 3 | 3.15 | 3.28 | 3.32 | 2.99 | 2.67 |  |  |
| QC Med | 1 | 6.37 | 5.66 | 5.36 | 5.23 | 5.02 |  |  |
| 6 | 2 | 6.45 | 5.87 | 5.89 | 6.29 | 5.28 | 8.0 | 3.1 |
|  | 3 | 6.26 | 6.11 | 6.19 | 5.73 | 5.47 |  |  |
| QC High | 1 | 12.25 | 12.30 | 11.97 | 12.52 | 12.22 |  |  |
| 14 | 2 | 14.80 | 15.28 | 15.07 | 14.90 | 13.85 | 9.3 | 2.9 |
|  | 3 | 15.46 | 13.50 | 12.83 | 14.06 | 12.89 |  |  |

**Quantifier versus qualifier peptide results**


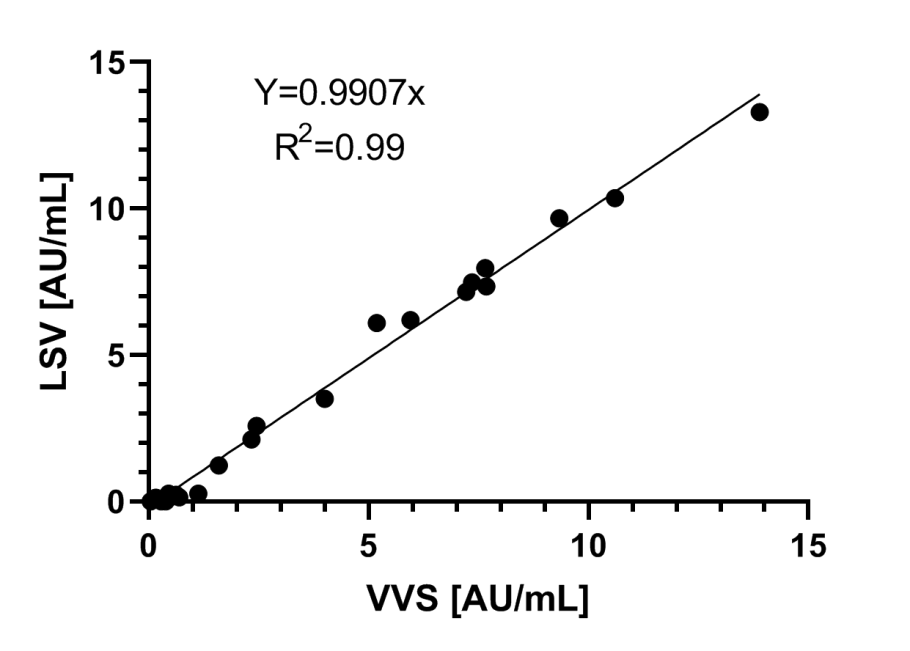


Figure S1: Result comparison of VVS peptide and LSV peptide for 30 EDTA plasma samples
